# Ectopic ACTH-dependent Cushing syndrome in 3 hospitalized patients: lessons learned from management with osilodrostat

**DOI:** 10.1210/jcemcr/luag213

**Published:** 2026-08-12

**Authors:** Hannah Ruggles, Emmy Yang, Nikita Mohan, Luis Borges Espinosa, Elizabeth Harris, Morgan Jones

**Affiliations:** Division of Endocrinology and Metabolism, Department of Internal Medicine, The University of North Carolina at Chapel Hill, Chapel Hill, NC 27514, USA; Division of Geriatric Medicine, Department of Internal Medicine, The University of North Carolina at Chapel Hill, Chapel Hill, NC 27514, USA; Division of Endocrinology and Metabolism, Department of Internal Medicine, The University of North Carolina at Chapel Hill, Chapel Hill, NC 27514, USA; Division of Endocrinology and Metabolism, Department of Internal Medicine, The University of North Carolina at Chapel Hill, Chapel Hill, NC 27514, USA; Division of Endocrinology and Metabolism, Department of Internal Medicine, The University of North Carolina at Chapel Hill, Chapel Hill, NC 27514, USA; Division of Endocrinology and Metabolism, Department of Internal Medicine, The University of North Carolina at Chapel Hill, Chapel Hill, NC 27514, USA

**Keywords:** ectopic ACTH-dependent Cushing syndrome, osilodrostat, hypercortisolism

## Abstract

Severe Cushing syndrome (CS) is a rare diagnosis with high mortality, which limits the ability of endocrinologists to gain experience delivering care. Here we describe our experience treating 3 cases of ectopic ACTH-dependent CS presenting in a single year. The initial diagnosis of each case was made during hospitalization resulting from complications of CS, implicating severe disease and delayed diagnosis. Our experience suggests that rapid initiation and titration of osilodrostat effectively treats hypercortisolism and improves outcomes.

## Introduction

Severe Cushing syndrome (CS) due to endogenous hypercortisolemia presents with metabolic effects of hyperglycemia, hypertension, weight gain, and hypokalemia, as well as complications including infection, thrombosis, psychoses, myopathy, and pathologic fractures. Clinical features of CS include hirsutism, acne, facial rounding, plethora, easy bruising, striae, and central adiposity. While ACTH-dependent hypercortisolemia is more frequently due to Cushing disease, ectopic ACTH syndrome (EAS) often presents with fulminant disease with more complications, intensive care unit (ICU) admissions, and higher mortality [1]. The highest mortality rate in CS occurs within the first 90 days of diagnosis, with advanced age and ectopic disease being risk factors [2]. High early mortality highlights the need for urgent treatment of hypercortisolism at the time of diagnosis.

Rapid control of hypercortisolism includes medical therapy with steroidogenesis inhibitors; bilateral adrenalectomy is often reserved for rescue therapy due to medical failure [3]. Osilodrostat is a newer steroidogenesis inhibitor (11β-hydroxylase) that can effectively control hypercortisolism [4, 5]. Emerging evidence suggests that osilodrostat outperforms other steroidogenesis inhibitors [6-8].

Existing literature describes osilodrostat use in outpatient settings, with limited data on initiation and titration in acutely ill hospitalized patients. Here, we present 3 cases of ectopic CS treated at a single center over the course of 8 months, providing a unique opportunity to gain experience using osilodrostat in the inpatient setting.

## Case presentation

### Case 1

A 67-year-old woman with a known metastatic pancreatic neuroendocrine tumor (under surveillance without treatment for 14 months) presented with dizziness, chest tightness, low back pain, shortness of breath, decreased appetite, urinary incontinence, and progressive lower extremity weakness. She was admitted for acute hypoxic respiratory failure from community-acquired pneumonia and hypokalemia. During hospitalization, she was found to have hypercortisolemia as well as multiple vertebral compression fractures. She was referred to our tertiary endocrine clinic for evaluation and management. Physical examination revealed cushingoid facies and wheelchair dependence.

### Case 2

A 68-year-old woman with hypertension, hyperlipidemia, and obesity presented with a year-long history of hypokalemia, new-onset type 2 diabetes mellitus, generalized weakness and frequent falls, recurrent urinary tract infections, and increasing anxiety. In the 2 months prior to CS diagnosis, she was admitted 3 times for lower extremity swelling and hypokalemia presumed secondary to heart failure exacerbation and diuretic use. During the fourth hospitalization for falls and weakness, she was diagnosed with CS and discharged with outpatient endocrine follow-up. She was readmitted 1 week later and transferred to our tertiary care center for endocrine consultation. Physical exam revealed hirsutism, ecchymosis, facial plethora, and cushingoid facies.

### Case 3

A 73-year-old woman with obesity, hypertension, type 2 diabetes mellitus, and hypothyroidism reported 2 months of progressive weakness, hyperglycemia, confusion, irritability, and weight gain. When she was hospitalized for hypertensive emergency and hypokalemia, hypercortisolemia was diagnosed. She was discharged to a rehabilitation unit with plans for outpatient endocrinology consultation but was referred to our tertiary care center due to rapid decline. Physical examination showed a dorsocervical fat pad, extensive ecchymosis, and new-onset atrial fibrillation. History revealed a similar constellation of signs and symptoms 4 years prior that spontaneously resolved after 8 months.

## Diagnostic assessment

### Case 1

Labs showed elevated serum cortisol, ACTH, and 24-hour urine free cortisol (UFC) (Table 1). An ectopic source of ACTH was attributed to a known pancreatic neuroendocrine tumor. Skeletal imaging revealed diffuse bone demineralization with bilateral sacral insufficiency fractures and multiple vertebral compression fractures (T11, L1-3, L5). Echocardiography demonstrated grade 1 diastolic dysfunction with reduced left ventricular global longitudinal strain (−12.7%).

**Table 1:** Diagnostic labs for Cushing syndrome

Case	Age, years/sex	ACTH Reference range 7.2-63 pg/mL (1.6-13.9 pmol/L)	Cortisol Reference range 5.2-22.4 µg/dL (143-618 nmol/L)	Cortisol following 8 mg DST suppression < 50% suggests ectopic source	24-hour urine free cortisol Reference range 3.5-45 µg/24 hours (9.7-124.2 nmol/24 hours)
1	67 Female	495 pg/mL (109 pmol/L)	83.9 µg/dL (2314.5 nmol/L)	ND	3890 µg/24 hours (10 736 nmol/24 hours)
2	68 Female	138 pg/mL (30.4 pmol/L)	41 µg/dL (1131 nmol/L)	36.1 μg/dL (995.9 nmol/L) 26% suppression	612 µg/24 hours (1689.1 nmol/24 hours)
3	73 Female	490 pg/mL (107.9 pmol/L)	86.4 µg/dL (2383.4 nmol/L)	68.2 µg/dL (1881.4 nmol/L) 21% suppression	2160 µg/24 hours (5961.6 nmol/24 hours)

Abbreviations: DST, dexamethasone suppression test; ND, no data.

### Case 2

Labs showed elevated cortisol, ACTH, and 24-hour UFC (Table 1). Inferior petrosal sinus sampling (IPSS) showed no significant central-to-peripheral ACTH gradient; however, prolactin control indicated inadequate catheter placement. Pituitary magnetic resonance imaging showed posterior sella focal hypointensity measuring 0.4 × 0.8 × 0.6 cm that was inconsistent with a pituitary adenoma. Octreotide scan was negative. Ga68-DOTATATE positron emission tomography (PET) could not be performed inpatient. Computed tomography (CT) imaging highlighted multiple vertebral compression fractures (L1-2, L4-5). Venous duplex revealed acute left lower extremity deep vein thrombosis. Echocardiography showed increased left ventricular wall thickness with normal ejection fraction and elevated left atrial filling pressure.

### Case 3

Labs once again showed elevated morning cortisol, ACTH, and 24-hour UFC (Table 1). A desmopressin stimulation test showed ACTH rise from 254 pg/mL (55.9 pmol/L) to 749 pg/mL (164.9 pmol/L). IPSS showed no central-to-peripheral ACTH gradient consistent with EAS; prolactin control confirmed proper cannulation on one side. Comprehensive imaging studies included pituitary magnetic resonance imaging, CT chest/abdomen/pelvis, octreotide scan, FDG PET/CT, and Ga68-DOTATATE PET/CT without source localization.

## Treatment

### Case 1

Spironolactone was started for refractory hypokalemia during hospitalization. Ketoconazole 200 mg twice daily (BID) was initiated as an outpatient 22 days following discharge. At day 24, the patient received her first dose of octreotide and gemcitabine chemotherapy. Ketoconazole was cautiously increased due to metastatic liver disease to 300 mg BID after 3 weeks. Outpatient management was complicated by infrequent follow-up, and thus additional steroidogenesis inhibitors were not initiated.

### Case 2

Spironolactone was initiated for refractory hypokalemia, and apixaban was initiated for deep vein thrombosis treatment; ketoconazole was contraindicated due to baseline liver enzyme elevation. Osilodrostat was started at 5 mg BID 9 days prior to transfer. Upon transfer to our center, osilodrostat was uptitrated over 4 weeks, achieving normal serum cortisol at a dose of 15 mg every morning and 20 mg every evening. During titration, the patient required initiation of hydrocortisone due to shock. Given the indeterminate IPSS results and the tumultuous clinical course, the patient underwent bilateral adrenalectomy.

### Case 3

Treatment with ketoconazole was started but discontinued after 1 week due to rising liver enzymes. Osilodrostat was initiated at 5 mg BID and uptitrated over 3 weeks: the dose was increased every 3 to 4 days. Biochemical control was achieved at a dose of 10 mg every morning and 15 mg every evening after 22 days, and physiologic hydrocortisone was subsequently initiated.

## Outcome and follow-up

### Case 1

Biochemical control was not achieved with medical management. The patient's course was complicated by recurrent admissions for acute hypoxic respiratory failure related to pneumonia and progression of vertebral compression fractures. The patient was transitioned to comfort care due to worsening respiratory status and progression of underlying disease; she died 98 days after EAS diagnosis.

### Case 2

Osilodrostat achieved biochemical control with serum cortisol 16.8 μg/dL (463.4 nmol/L) prior to definitive therapy with bilateral adrenalectomy. The patient showed clinical improvement but died 11 days after bilateral adrenalectomy and 89 days after CS diagnosis due to cardiac arrest.

### Case 3

Biochemical control was achieved on osilodrostat with a 24-hour UFC of 25 μg/24 hours (68.9 nmol/24 hours) and serum cortisol of 10.4 μg/dL (286.9 nmol/L) at day 22. Significant clinical improvements included 20-pound weight loss, reduction in insulin requirements, reduction of antihypertensive medications, and normalization of potassium levels. She was discharged to a skilled rehabilitation facility on medical therapy.

## Discussion

Delayed diagnosis of CS is common, with an average time to diagnosis of 2 to 3 years across etiologies [9, 10]. Although EAS is often recognized earlier due to fulminant complications, the average time to diagnosis still ranges from 6 to 14 months [1, 10]. Endocrine involvement is therefore nearly universally delayed and the impetus for urgent treatment is high, but even the practiced endocrinologist must navigate multiple treatment options as we did over the course of these 3 cases.

Our 3 cases of CS had biochemically severe disease with UFC greater than 10 times the upper limit of normal (Table 1). All patients were diagnosed during hospitalization for complications of previously unrecognized hypercortisolism (Table 2), and 2 required ICU-level care. Two of our 3 patients died within 98 days of biochemical diagnosis, consistent with the high mortality rates reported in patients with severe CS (Table 2) [1, 2, 11]. The burden of complications in our cohort, including refractory hypokalemia, hyperglycemia, venous thromboembolism, vertebral fractures, cardiomyopathy, and recurrent infections (Table 3), underscores the multiorgan toxicity of sustained hypercortisolism. Echocardiographic abnormalities were observed in 2 patients, aligning with prior studies demonstrating left ventricular dysfunction in CS [12].

**Table 2:** Treatment and outcomes

Case Age, years/sex	Admitting diagnosis	Time from diagnosis to treatment*^a^*	Time to death/discharge from diagnosis	Time to biochemical control after osilodrostat initiation	Sequence of endocrine treatment
1 67 Female	Acute hypoxic respiratory failure and hypokalemia	29 days	98 days, died	NA (Never started osilodrostat)	Ketoconazole, octreotide
2 68 Female	Lower extremity edema and recurrent falls	35 days	89 days, died	44 days	Osilodrostat, bilateral adrenalectomy
3 73 Female	Progressive weakness and hypertensive emergency	31 days	77 days, discharged	22 days	Ketoconazole, osilodrostat

^*a*^Time from first biochemical evidence of disease until initiation of cortisol-directed medical therapy.

**Table 3:** Complications related to Cushing syndrome

Case	Hypokalemia	Hypertension	Hyperglycemia	Deep vein thrombosis	Fracture	CV*^a^*	Psychiatric disorder	Sepsis/infection	Myopathy
1	X		X		X	X	X	X	X
2	X	X	X	X	X	X	X	X	X
3	X	X	X			X	X	X	X

Echocardiogram findings of left ventricular dysfunction in cases 1 and 2; atrial fibrillation in case 3.

^*a*^Cardiovascular complication.

In all our patients, therapy was not initiated until 29 to 35 days after biochemical diagnosis. Therapy was delayed by limited endocrine availability as well as completion of the diagnostic workup. Lavoillotte et al showed high specificity for EAS when UFC is ≥20 times the upper limit of normal, but even in their proposed diagnostic algorithms, they do not eliminate the recommendation for IPSS in the absence of CT evidence of a tumor; further research in this area is needed to expedite therapy [13]. As seen in case 1, attempts for outpatient management led to failure to initiate effective medical therapy. Biochemical control was achieved in 2 patients treated with osilodrostat, with the most rapid normalization occurring in case 3 following accelerated inpatient titration. However, as illustrated by case 2, biochemical remission did not immediately reverse accumulated cardiovascular risk; sudden cardiac death occurred despite biochemical control of CS.

Osilodrostat is a potent 11β-hydroxylase inhibitor that has proven efficacious in clinical trials and is US Food and Drug Administration–approved for CS [4, 5]. Several other agents were considered or attempted, each with notable barriers to use. Ketoconazole and levoketoconazole demonstrate similar efficacy in inhibiting multiple steps in the steroidogenesis pathway, and ketoconazole has the additional benefit of being both cost-effective and readily available [3, 14, 15]. However, their use was limited in our patients due to hepatotoxicity and drug-drug interactions. Metyrapone, which inhibits 11β-hydroxylase, is approved for treatment of CS in several European countries, but prescribing in the United States is constrained by a US Food and Drug Administration–approved indication limited to diagnostic testing for adrenal insufficiency rather than CS treatment [3, 14]. In case 1, insurance approval for metyrapone was sought but denied, and out-of-pocket costs were prohibitive. Etomidate, which inhibits 11β-hydroxylase and cholesterol side-chain cleavage enzyme, is an effective adrenolytic agent and the only available intravenous option [16]. Etomidate infusion can achieve rapid control of hypercortisolism and serve as a bridge to oral therapies or adrenalectomy; Dzialach et al described minimal sedative effects and safe use outside the ICU setting with a low-dose infusion [17]. Etomidate infusion was not available on our formulary. Mifepristone acts by blocking the glucocorticoid receptor and does not lower cortisol levels, interfering with biochemical monitoring of response. Furthermore, mifepristone's side effects, including hypokalemia, hypertension, and fluid retention, limit its utility in severe CS cases [3]. Given these constraints, our selection of osilodrostat was pragmatic and effective in controlling cortisol levels.

Our center's experience with osilodrostat illustrates both its efficacy and the practical challenges of inpatient implementation. The literature describes multiple approaches to osilodrostat use in severe CS: titration without combination glucocorticoid therapy, block-and-replace regimen upfront, or initial titration followed by block-and-replace secondarily [8, 18]. Both cases 2 and 3 utilized the initial titration followed by block-and-replace strategy. We used higher initial doses of 10 mg daily in both cases given the severity of hypercortisolism following the experience of Dormoy et al [8]. Osilodrostat dose adjustments were made by following serum cortisol levels; both patients required glucocorticoid replacement for adrenal insufficiency during the treatment course. Inpatient admission allows for close monitoring for medication side effects including hypokalemia, QT prolongation, and hypocortisolism, as well as ongoing management of CS-induced comorbidities such as hypertension and hyperglycemia. In sum, osilodrostat achieved biochemical control in both cases, and when rapidly deployed as monotherapy (case 3), was associated with patient survival.

High clinical suspicion for CS is paramount as delayed diagnosis remains the most significant barrier to timely treatment. All our patients presented with a constellation of findings including hypokalemia, hyperglycemia, cardiovascular complications, psychiatric disturbances, infections, and severe myopathy rendering them nonambulatory, which should have suggested hypercortisolism. Urgent endocrine consultation is essential when CS is suspected so as not to delay treatment. In our series, osilodrostat delivered rapid biochemical control and appeared to contribute to improved outcomes in our sole surviving patient.

## Learning points

- Patients admitted with features suspicious for CS should have an urgent endocrinology consultation.
- Osilodrostat achieves rapid control of hypercortisolism when titrated aggressively.
- Inpatient admission is essential to allow adequate monitoring for rapid titration of osilodrostat.

## Data Availability

Data sharing is not applicable to this article as no datasets were generated or analyzed during the current study.
